# *Caenorhabditis elegans* Slow Killing recapitulates chronic *Pseudomonas aeruginosa* infection and reveals mediators of host-pathogen interactions

**DOI:** 10.3389/fmicb.2026.1990701

**Published:** 2026-10-21

**Authors:** Sadie Gaskins, Liyang Zhang, Donghoon Kang, Qi Xu, Natalia V. Kirienko

**Affiliations:** 1 Department of BioSciences, Rice University, Houston, TX, United States; 2 Department of Bioengineering, Rice University, Houston, TX, United States

**Keywords:** *Caenorhabditis elegans*, colonization, Liquid Killing, *Pseudomonas aeruginosa*, RNA sequencing, Slow Killing, transcriptomics

## Abstract

*Pseudomonas aeruginosa* is a leading cause of healthcare-associated infections, especially in immunocompromised patients and individuals with cystic fibrosis (CF). *P. aeruginosa* commonly causes chronic infections within the respiratory tracts of patients with CF. These infections are marked by a shift from the production of acute virulence factors (secreted toxins) to colonization (biofilm formation) and persistence within the host. To better understand the transcriptomic changes that occur during this shift within the context of host-pathogen interactions, we took advantage of a whole-organism model system. Using an established *Caenorhabditis elegans – P. aeruginosa* colonization model, we identified genes differentially regulated during establishment of the infection of the *C. elegans* intestine. By comparing this transcriptomic signature to those from *P. aeruginosa* from CF sputum samples and an *ex vivo* porcine lung biofilm model, we demonstrated that Slow Killing partially recapitulates chronic lung infection. Notably, we observed the upregulation of lipid A and exopolysaccharide (Pel, alginate) biosynthetic genes within the nematode host and downregulation of acute virulence pathways including quorum-sensing, phenazine production, toxin secretion, and siderophore-mediated iron uptake. Further characterization of these differentially-regulated genes revealed key bacterial pathways, including quorum-sensing and the PhoPQ regulatory network, that impacted host survival during infection. These results demonstrate that the *C. elegans* Slow Killing model is a highly accessible tool for studying host-pathogen interactions and benchmark it against clinical samples.

## 1 Introduction

*Pseudomonas aeruginosa* is a Gram-negative, opportunistic human pathogen that causes a wide-range of infections, from hospital-acquired ventilator-associated pneumonia and bloodstream infections to chronic lung infections in persons with cystic fibrosis (CF), where it is the predominant cause of morbidity and mortality (Jurado-Martín et al., 2021; Moradali et al., 2017; Letizia et al., 2025). Clinical isolates of *P. aeruginosa* often exhibit substantial metabolic plasticity, readily adapting to diverse environment, complicating their removal from health care settings (Moradali et al., 2017). These strains are also often intrinsically resistant to several classes of antibiotics and readily evade host immune responses, further contributing to the urgent need for new treatments (Moradali et al., 2017; Qin et al., 2022).

Acute *P. aeruginosa* infections are characterized by the production of proteases such as the elastase LasB and protease PrpL (protease IV), that can disrupt host proteins that compose the extracellular matrix or modulate immune responses (Prasad et al., 2020; Bainbridge and Fick, 1989; T.-H. Kim et al., 2021), and the secretion of cytotoxins such as ExoU, ExoS, exotoxin A, hemolytic phospholipase C, and rhamnolipids (Moradali et al., 2017; Qin et al., 2022; Jurado-Martín et al., 2021; Casilag et al., 2016; Saint-Criq et al., 2018; Hauser, 2009; Yahr et al., 1996; Xu et al., 2024; Lovewell et al., 2014; Barker et al., 2004; García-Contreras et al., 2020). Most of these virulence factors are regulated by small molecules (e.g.: N-acyl homoserine lactones produced by the LasRI and RhlRI systems, *Pseudomonas* quinolone signal (PQS), ferripyoverdine, cyclic diguanylate monophosphate (c-di-GMP)) that mediate inter- and intracellular signaling (Conaway et al., 2025; Qin et al., 2022; Moradali et al., 2017; Jurado-Martín et al., 2021).

*P. aeruginosa* is also capable of causing chronic infections marked by a shift towards a biofilm-associated phenotype and characterized by the upregulation of genes required for the production of extracellular polymeric substances and other processes, including lipid A biosynthesis (Bjarnsholt et al., 2010; McPhee et al., 2006; Schurek et al., 2009). Chronic infections are also associated with the loss of acute virulence factors often driven by the inactivation of quorum-sensing regulators (e.g., LasR) (Smith et al., 2006; Flores-Vega et al., 2026; Hall et al., 2022).

We previously surveyed *P. aeruginosa* isolates from pediatric patients with cystic fibrosis for the production of acute virulence factors, the major siderophore pyoverdine and rhamnolipids, that are important for pathogenesis against *Caenorhabditis elegans*, murine macrophages, and human bronchial epithelial cells (Xu et al., 2024; Kang and Kirienko, 2021; Kang et al., 2019, 2024). Consistent with the acute-to-chronic switch in the lungs of CF patients (Jurado-Martín et al., 2021), ~30% of strains exhibited either very low or no pyoverdine production and more than half of the isolates lost the ability to produce rhamnolipids (Xu et al., 2024; Kang et al., 2019). However, it remained unclear how this switch occurs within the host and which host or pathogen factors drive this change.

In this study, we took advantage of the nematode host *C. elegans* to determine how *P. aeruginosa* responds to the environment inside of the host during colonization. There are at least two major models established for investigating *P. aeruginosa* pathogenesis in *C. elegans.* In the agar-based Slow Killing (SK) model, the worm is exposed to a lawn of *P. aeruginosa*; the pathogen exerts virulence by colonizing the intestinal epithelium (Tan et al., 1999a), and colonization-deficient mutants exhibit attenuated virulence (Tan et al., 1999a; Tan et al., 1999b). In this model, the host resists bacterial infection through the p38 MAPK/PMK-1 innate immune pathway (Kim et al., 2002). We previously modified this model to establish a liquid-based pathogenesis assay (Liquid Killing, LK), where *P. aeruginosa* exerts virulence through the production of pyoverdine and secreted toxins without colonizing the host, while colonization is strongly impeded (Kang et al., 2018a; Kang et al., 2019; Zhang et al., 2023; Kirienko et al., 2013). Deprivation of host iron by pyoverdine elicits a distinct host defense response through the ethanol and stress response element (ESRE) mitochondrial surveillance pathway (Tjahjono and Kirienko, 2017; Kang et al., 2018a).

We utilized Slow- and Liquid- Killing pathosystems in addition to the no-nematode media controls to characterize the transcriptomic profiles of *P. aeruginosa* from inside of the host. Unfortunately, very low-level of bacterial colonization precluded us from obtaining quantifiable data for the LK condition. However, the yield of *P. aeruginosa* RNA from the SK condition was comparable to that of CF sputum samples. When we compared the *P. aeruginosa* transcriptomic signatures from the nematode SK infection model to those from human sputum samples or an *ex vivo* porcine lung biofilm model, we found significant similarities between *C. elegans* SK and chronic lung infection. *P. aeruginosa* downregulated acute virulence genes within the nematode host while upregulating genes required for biofilm formation and lipid A synthesis. Several of these differentially-expressed genes, such as those involved in quorum-sensing or part of the PhoPQ two-component system, were important for *P. aeruginosa* virulence during SK. Our findings identify specific pathogenesis-related genes and pathways for which *C. elegans* model closely recapitulates *P. aeruginosa* lung infections.

## 2 Materials and methods

### 2.1 Preparation of *P. aeruginosa* and *C. elegans* samples

To prepare *P. aeruginosa* for infection, *P. aeruginosa* PA14 (Mathee, 2018) was inoculated into 5 mL of LB broth, followed by incubation at 37 °C with shaking for 14–16 h. The overnight culture was seeded onto SK (Slow Killing) plates (Anderson et al., 2018), which were then incubated at 37 °C for 24 h and at 25 °C for another 24 h.

The temperature-sensitive sterile *glp-4(bn2)* worms (Beanan and Strome, 1992) were placed onto 10 cm Nematode Growth Medium (NGM) agar plates with a lawn of *Escherichia coli* OP50 (“superfood” plates) at a density of 10,000 L1 larvae per plate and incubated at room temperature overnight. After that, worms were transferred to an incubator set at 25 °C for 48 h until L4/young adult stage. Worms were washed off from the superfood plates using S Basal buffer (5.85 g/L NaCl, 1 g/L K_2_HPO_4_, 6 g/L KH_2_PO_4_) and collected into 50 mL conical tubes, with three subsequent washes using 50 mL of S Basal per wash.

Four conditions were used for RNA sequencing: (1) *P. aeruginosa* grown *in vitro* on agar medium, (2) *P. aeruginosa in vivo* on agar medium (colonized the nematode host during SK), (3) *P. aeruginosa* grown *in vitro* in LK medium, and (4) *P. aeruginosa in vivo* during LK. For the agar growth condition, PA14 was resuspended from one 10 cm SK plate in 5 mL of S Basal. For the SK condition, approximately 2,500 L4/young adult worms were transferred onto two 10 cm PA14 SK plates. For the liquid growth condition, PA14 was inoculated to an initial density of O. D. 600 nm 0.03 in LK medium (Anderson et al., 2018). For LK conditions, 100 mL of LK medium inoculated with PA14 was mixed with L4 worms, and 2 mL of this worm suspension was added to each well of eight 6-well plates, resulting in ~5,000 worms per well. Agar and 6-well plates with *C. elegans* were incubated at 25 °C for eight hours. *P. aeruginosa* and worm samples were washed in S Basal, pelleted, resuspended in RNAlater solution (Thermofisher Scientific), and stored at −80 °C prior to RNA extraction.

### 2.2 RNA sequencing

RNA from *C. elegans*/*P. aeruginosa* samples were extracted as previously described (Cornforth et al., 2020). In brief, RNAlater solution was removed after centrifugation and samples were resuspended in TE (10 mM Tris, 1 mM EDTA) buffer with 1 mg/mL lysozyme. Tissue/cells were homogenized by bead beating and enzymatic lysis. RNA was isolated by phenol-chloroform purification using RNA-Bee/Stat60 reagent (AMSBIO), precipitated in isopropanol and linear acrylamide, and resuspended in nuclease-free water. RNA samples underwent DNase digestion (Promega) and were analyzed on an Agilent 2,100 Bioanalyzer for RNA integrity. Ribosomal RNA depletion and fragmentation was performed on all samples using a Qia Seq FastSelect kit (Qiagen) with 5S/16S/23S depletion reactions for *in vitro* (agar, liquid) samples and 5S/16S/23S and HMR depletion reactions for *in vivo* (SK, LK) samples. DNA libraries for sequencing were prepared using a NEBNext Small RNA Library Prep Kit for Illumina (New England Biolabs) according to manufacturer guidelines. Approximately ~150 bp libraries were obtained by running libraries on a 6% polyacrylamide gel and cutting out DNA fragments visualized with a SYBR Gold nucleic acid stain. DNA libraries were solubilized in gel elution buffer, precipitated in 100% ethanol with linear acrylamide and 0.1 M sodium acetate, and resuspended in nuclease-free water. Quality and concentration of libraries were analyzed on an Agilent 2,100 Bioanalyzer. Next generation sequencing was performed at the Parker H. Petit Institute for Bioengineering and Bioscience, Georgia Tech on a NextSeq system (Illumina). *In vitro* samples (liquid, agar) were sequenced at 15 million reads per sample. SK samples were sequenced at 65 million reads each and LK samples were sequenced at 100 million reads each. Bioproject Accession number GSE346153.

### 2.3 Differentially-expressed genes (DEG) analysis

Short reads from Illumina sequencing were concatenated for each sample. Reads were filtered for quality and adapters were trimmed using TrimGalore. Ribosomal RNA was removed by SortmeRNA (Kopylova et al., 2012). Reads were first aligned to the *C. elegans* N2 genome (NC_003279.8) using Bowtie2 and unaligned reads (from *P. aeruginosa*) were saved for downstream analysis. Remaining reads were aligned to the PAO1 (NC_002516.2) or PA14 (NC_008463.1) reference genomes using Bowtie2. Mapped reads per gene were quantified by FeatureCounts. Differentially-expressed genes (DEGs) were identified using iDEP2.0 (Ge et al., 2018). For pre-process filtering, genes without at least 0.5 counts per million in at least 6 libraries (comparing two conditions, 3 libraries each) were removed and data were transformed using EdgeR. Threshold for DEGs were set at an adjusted *p*-value (FDR) < 0.05 and minimum fold-change of log_2_1.5. For reads aligned to PAO1, DEG pathways were identified by gene ontology (GO biological processes) or STRING analysis.

### 2.4 Accuracy score (AS_2_) calculation

AS_2_ values were calculated following the methods described in previous publications (Cornforth et al., 2020; Lewin et al., 2023). Briefly, RNAseq data from our model and publicly available data from human CF sputum samples were normalized using variance stabilizing transformation (VST). All genes were annotated using TIGRFAM to assign metadata, main roles, sub-roles, and functions. AS_2_ was calculated based on the percentage of genes with a z-score within an absolute value of 2. The mean from our model, along with the mean and standard deviation from human isolates, was used to calculate the z-scores. The sunburst plots were generated using code adapted from the GitHub repository: https://github.com/glew8/PA_ModelAccuracy (Lewin et al., 2023).

### 2.5 Nematode colonization assay

*C. elegans* colonization by PA14 in-frame deletion or transposon insertion mutants were measured via fluorescence microscopy. *P. aeruginosa* strain PA14 (wild-type) and its transposon mutants were all obtained from the UCBPP-PA14 transposon mutant library (Liberati et al., 2006). In-frame deletion mutants (Δ*phz*, Δ*pelA*) were sourced from previous studies (Dietrich et al., 2006; Kuchma et al., 2007). The lasR mutant was constructed in this study through two-step allelic exchange (Hmelo et al., 2015). Strains were transformed with a plasmid (pRO1600 origin of replication, *dsRed* under *lac* promoter, ampicillin resistance cassette) that allows for constitutive *dsRed* expression (Kirienko et al., 2013) by chemical transformation and selection on LB agar with 300 μg/mL carbenicillin. The chemical transformation procedure was modified from a previously established protocol (Chuanchuen et al., 2002). Briefly, the overnight culture of *P. aeruginosa* was first incubated on ice for 30 min and collected by centrifugation. The cell pellet was washed with 1 mL of pre-chilled 0.1 M MgCl₂, collected by centrifugation, and resuspended in 100 μL of pre-chilled 0.1 M CaCl₂. The cell suspension was incubated on ice for 20 min, followed by the addition of 1 μL of dsRed plasmid and incubation on ice for 1 h. The cells were heat shocked at 42 °C for 1 min and were immediately placed on ice for 2 min. The cells were recovered in 1 mL of pre-warmed SOC medium and incubated at 37 °C with shaking for 2 h. The recovered cells were subsequently spread onto LB agar containing 300 μg/mL carbenicillin. After overnight incubation, individual colonies were screened for fluorescence and inoculated into LB broth containing 300 μg/mL carbenicillin for further confirmation. Transformed strains were cultured overnight in LB broth with 300 μg/mL carbenicillin at 37 °C with shaking for 14–16 h. The overnight culture was spread onto SK plates, incubated for 24 h at 37 °C, and then for another 24 h at 25 °C. Young adult *glp-4*(*bn2*) worms were picked onto the bacterial lawn and left for 24 h infection at 25 °C. At least 40 worms per condition were picked into a microcentrifuge tube with 1 mL S Basal buffer containing 3 mg/mL levamisole and 0.01% Tween-20 (v/v) and washed twice. Worms were imaged on glass slides with agarose pads under a Zeiss Axio Imager M2 fluorescence microscope. Image analysis was performed via Matlab.

## 3 Results

### 3.1 *P. aeruginosa* exhibits a distinct transcriptomic signature within the nematode host

To assess the impact of growth environment and host colonization on the transcriptome of *P. aeruginosa*, the laboratory strain PA14 was grown on SK agar with or without *C. elegans* present (i.e., bacteria alone or bacteria plus host). *P. aeruginosa* strain PA14 was also grown in LK medium with or without *C. elegans*. Total RNA was harvested and sequenced from either bacteria or worms and bacteria for each condition (Figure 1A). While aligning bacterial RNA read sequences, we determined that poor colonization in LK medium precluded harvesting sufficient bacterial RNA for robust analysis, so this sample was excluded from further analysis. This was not unexpected, as previous findings indicated that there was little to no colonization of the host during exposure of *C. elegans* to *P. aeruginosa* in LK conditions (Kirienko et al., 2013).

**Figure 1:**
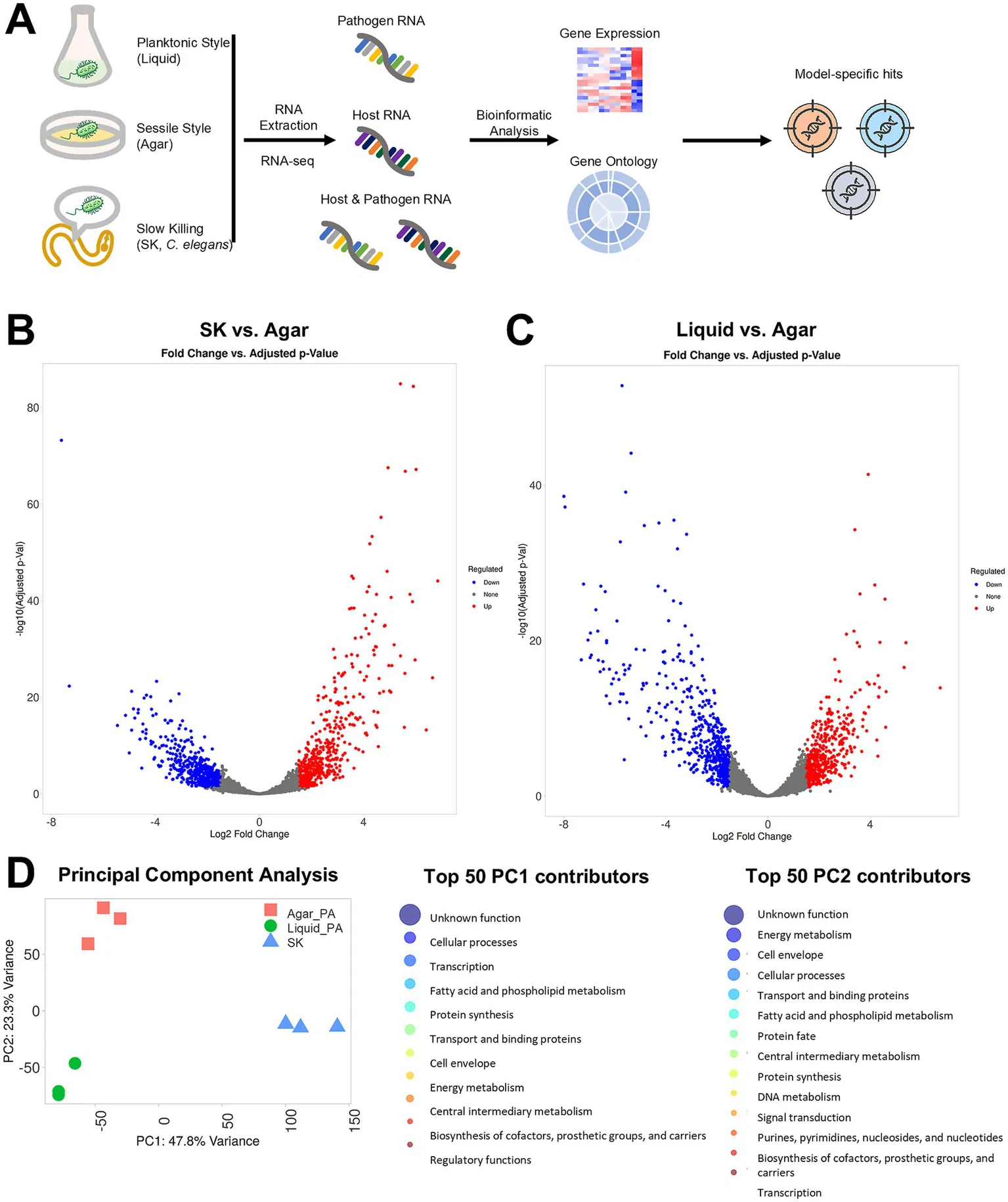
*P. aeruginosa* exhibits a distinct transcriptomic profile during *C. elegans* colonization. **(A)** Experimental design for RNA sequencing. **(B,C)** Volcano plot of differentially-expressed genes (DEGs) derived from feature count data from sequencing reads aligned to the PAO1 reference genome. **(B)** DEGs from *P. aeruginosa* that colonized *C. elegans* (SK) compared to bacteria grown on agar. **(C)** DEGs from *P. aeruginosa* grown in liquid medium compared to bacteria grown on agar. **(D)** Principal component analysis of *in vitro* (agar, liquid) and *in vivo* samples based on RNAseq analysis. Bubble plots show main pathways contributing to principal component scores.

For the remaining samples, *C. elegans*-derived transcripts were removed and remaining reads were aligned to the PAO1 reference genome. Transcriptomes were examined to identify differentially expressed genes (DEGs) in each condition (Figures 1B,C). Principal component analysis appropriately clustered gene expression profiles from different samples (Figure 1D), indicating good separability of the biological signals from the test conditions. This analysis also indicated that bacterial samples in the absence of the predator-host *C. elegans* were more similar to each other than either the sample of bacteria on the plate that were being consumed by, or colonizing, the nematode host (Supplementary Tables S1, S2). Gene ontology (GO) analysis of the factors driving these differences largely highlighted genes with unknown function, with some smaller categories also present, including transcription, fatty acid and phospholipid metabolism, and protein synthesis (Figure 1D). Similarly, the genes separating the two conditions only including bacteria were heavily weighted for genes with unknown function, while genes associated with energy metabolism, the cell envelope, and transport and binding proteins were also differentially-expressed between these conditions.

### 3.2 *C. elegans* Slow Killing partially recapitulates *P. aeruginosa* chronic lung infection

Previous studies demonstrated that growing *P. aeruginosa* in simulated host conditions, such as synthetic cystic fibrosis medium (SCFM), can closely recapitulate transcriptional events during chronic lung infection (Cornforth et al., 2018; Lewin et al., 2023). Using available transcriptomic data from CF sputum samples (Lewin et al., 2023), we examined whether transcriptional data from *P. aeruginosa* induced by SK is similar to those from sputum. To quantitatively assess similarities across samples, Accuracy Scores (AS_2_) were calculated (Cornforth et al., 2020). These scores measure the proportion of genes with expression levels that fall within two standard deviations of the reference dataset, with higher AS_2_ values indicating greater similarity.

To evaluate how well the nonhuman models capture features of *P. aeruginosa* infection in humans, we first compared the combined transcriptomic profiles from all conditions (*P. aeruginosa* grown *in vitro* on agar or in liquid medium, or *in vivo* during SK) with those of *P. aeruginosa* from CF sputum samples. This comparison yielded an AS_2_ score exceeding 80% (Figure 2A), indicating that the nonhuman models are informative for studying infection despite their limitations. Because *C. elegans* is extensively used in infection research (Martinez et al., 2026; Mahajan-Miklos et al., 1999; Kim et al., 2021; Kang et al., 2018a; Feinbaum et al., 2012; Tan et al., 1999a), we next specifically investigated how *P. aeruginosa* colonization during SK reflects human infection. We noted that, while the number of reads per gene were substantially lower for these samples than other bacterial-only samples, likely due to the co-isolation of host RNA, the sequencing depth and coverage were still comparable to those from human sputum samples (Figure 2B) (Lewin et al., 2023). Compared with CF sputum samples, the transcriptomic profiles of *P. aeruginosa* recovered from colonized *C. elegans* showed substantial similarity, with AS_2_ scores exceeding 75% (Figure 2C). This suggests that *C. elegans* colonization partially recapitulates transcriptional features of human chronic lung infection. Because both SK-infected *C. elegans* and CF sputum represent host-associated environments, we reasoned that they impose overlapping selective pressures on *P. aeruginosa*. To identify bacterial functions associated with this overlap, we used TIGRFAM annotations to analyze genes with similar expression profiles in SK and CF sputum, excluding genes that also met the similarity criterion in the bacteria-only agar or liquid conditions. The resulting gene set included functions related to energy metabolism, protein fate, and transport and binding (Figure 2D). These findings indicate that the *C. elegans* SK model reproduces a subset of *P. aeruginosa* transcriptional responses observed during human infection.

**Figure 2:**
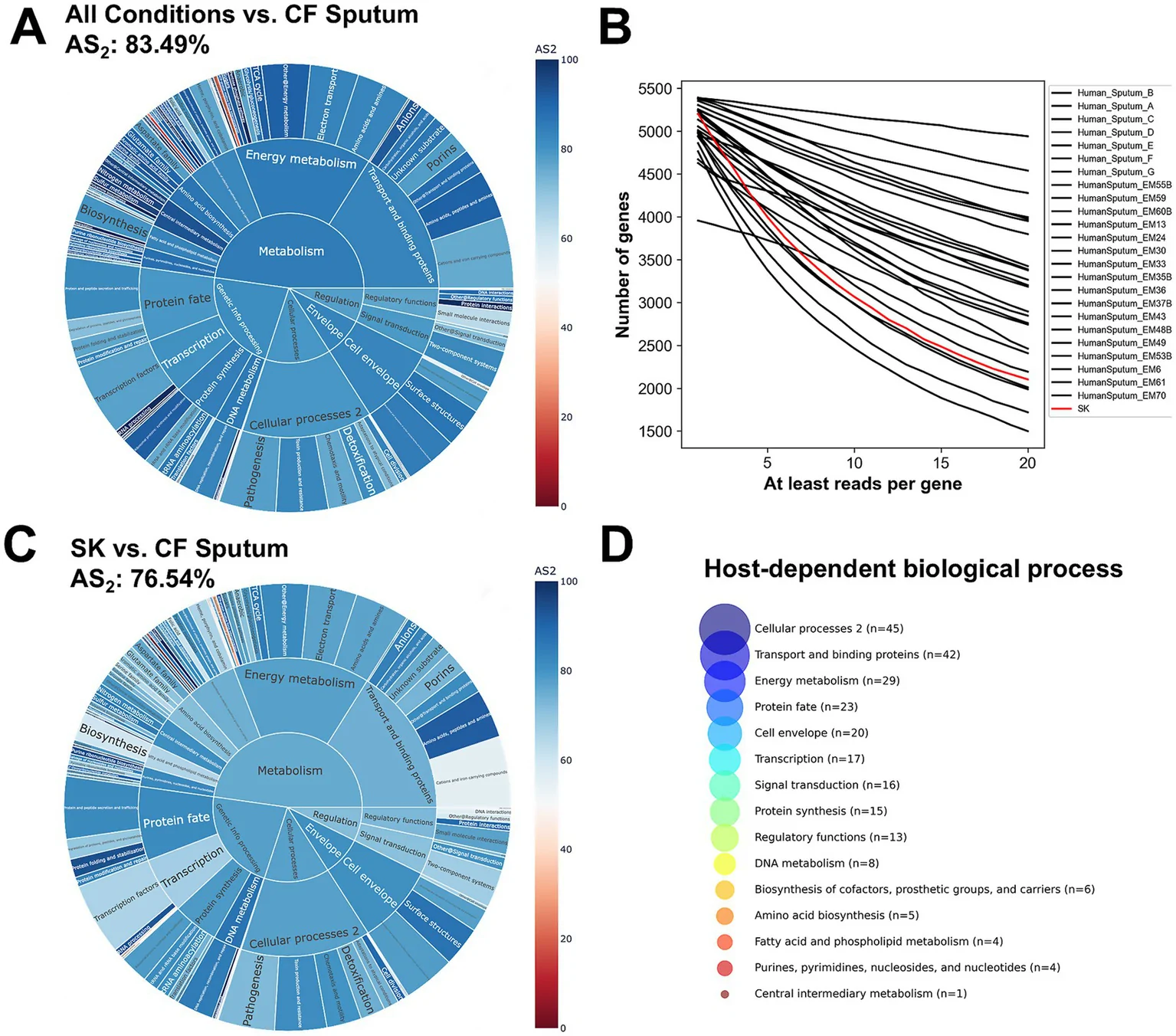
Bacterial transcriptome within the nematode host partially recapitulates those from CF sputum samples. **(A)** Sunburst diagram representing AS_2_ scores comparing the combined transcriptomic profiles from all experimental conditions (*in vitro* agar or liquid, *in vivo* SK) to those from human CF sputum samples. The blue-to-red color scale indicates high to low similarity, respectively. **(B)** Sequencing coverage and depth (number of reads per gene) from RNA sequencing for human sputum samples (black) and SK (red). Reads from CF sputa were acquired from a previous study (Lewin et al., 2023). **(C)** Sunburst diagram representing AS_2_ scores comparing the SK condition to CF sputum samples. **(D)** TIGRFAM functional categories of genes with similar expression profiles in SK and CF sputum samples (C), excluding genes that also showed similar expression in the bacteria-only agar or liquid conditions.

Next, to specifically examine how closely pathogenesis-associated genes in the *P. aeruginosa* transcriptomic profiles collected across our experimental conditions recapitulate the differential expression patterns observed in pathogenesis-associated genes in CF sputum, we examined AS_2_ scores for pathogenesis-associated genes. Pathogenesis-associated genes were identified from previously established TIGRFAM functional annotations under the main role “Cellular processes.” (Haft et al., 2001). We compared genes differentially regulated in *P. aeruginosa* grown on SK agar medium (agar), in LK medium (liq), and in bacteria that had colonized *C. elegans* (SK) to CF sputum individually (Figures 3A–C). These comparisons provided insight about which transcriptional aspects of *P. aeruginosa* pathogenesis observed in CF sputum were recapitulated in each of our experimental conditions, and allowed us to identify any differentially regulated genes that were unique to a specific condition. Each condition exhibited AS_2_ > 70% when compared to CF sputum. Of the remaining 126 genes outside the AS_2_ cutoff, 76 genes were shared across the three conditions from this study (Figure 3D). Fourteen genes were identified as unique to SK (Supplementary Table S3) and were significantly downregulated compared to *in vitro* growth on agar (Supplementary Table S1). The majority of these genes have been associated with *P. aeruginosa* virulence (quorum-sensing, phenazine production, biofilm formation, toxin secretion). For this reason, we systematically examined how host colonization impacted expression of virulence genes.

**Figure 3:**
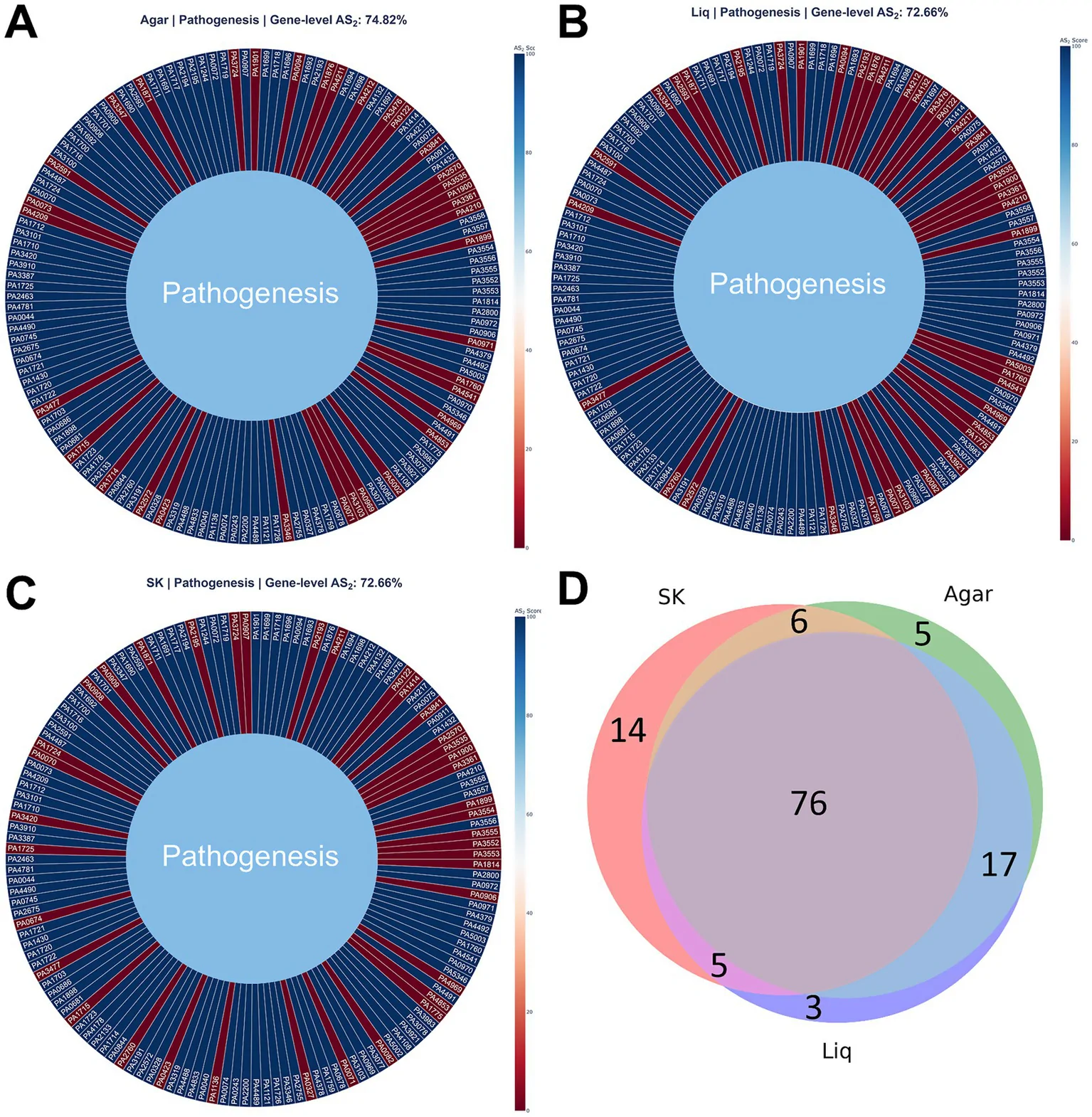
Identification of pathogenesis genes associated with *C. elegans* infection. **(A–C)** AS_2_ scores comparing the expression of pathogenesis-related genes (Figure 2) between *P. aeruginosa* from CF sputum samples and *P. aeruginosa* from growth on agar **(A)**, growth in liquid **(B)**, or host colonization during SK **(C)**. **(D)** Venn diagram showing the number of shared and unique pathogenesis genes among the three experimental conditions for genes outside the AS_2_ cutoff (differentially regulated in the experimental conditions when compared to human sputum samples). Genes unique to SK are listed in Supplementary Table S3.

To validate our analytical approach, we used gene ontology (GO) and protein–protein interaction analysis (STRING) to query the function of DEGs between bacteria grown on SK agar medium and LK medium. Based on our established model of *C. elegans* LK, which relies on production of pyoverdine, but not pyochelin, for *P. aeruginosa* pathogenesis (Kirienko et al., 2013), we expected to observe increased expression of pyoverdine biosynthetic genes in LK medium. We also anticipated increased expression of motility genes in the liquid environment. We identified several virulence functions, such as siderophore-dependent iron uptake and biofilm formation, that were upregulated in liquid medium (Supplementary Table S4). Manual inspection of virulence genes revealed significant upregulation of several iron uptake pathways such as heme (Has, Phu systems) uptake and pyoverdine biosynthesis, including genes encoding PvdS-regulated toxins (the translational inhibitor exotoxin A and the protease PrpL), but not pyochelin biosynthesis (Supplementary Table S5). Furthermore, we observed upregulation of the type II secretion system, which is responsible for the secretion of exotoxin A and PrpL, among other secreted virulence factors (Kang et al., 2021). The association of the liquid environment with the STRING pathway for anti-sigma factor antagonist activity and biofilm formation was likely due to upregulation of the *Pseudomonas* quinolone signal (PQS) quorum-sensing system and type IV pilus biogenesis genes that simultaneously regulate *P. aeruginosa* cell aggregation and motility (Ha et al., 2011; Kang et al., 2018b; O’Toole and Kolter, 1998; Kang et al., 2018a).

We performed similar analyses for DEGs from *P. aeruginosa* that colonized the nematode host during SK compared to bacteria grown on agar. Since *P. aeruginosa* colonizes *C. elegans* during SK, we anticipated upregulation of genes that would help the transition from motile to sessile state. We also expected to see downregulation of genes associated with acute virulence, such as siderophore production. GO and STRING analysis indicated that genes involved in lipid A biosynthesis and biofilm formation were upregulated during colonization while genes required for siderophore production were downregulated (Supplementary Table S4). In-depth examination of specific virulence genes confirmed the upregulation of the lipid A biosynthetic locus (*arn*), the two-component systems *phoPQ* and *pmrAB* (which regulate lipid A biosynthesis and colistin resistance), and the *feoAB* ferrous iron uptake system (Table 1) (McPhee et al., 2006; Schurek et al., 2009; Miller et al., 2011). Genes involved in exopolysaccharide synthesis (Pel, alginate) that are required for biofilm formation were upregulated within the nematode host. In contrast, global downregulation of acute virulence factors, including genes involved in siderophore biosynthesis (pyoverdine, pyochelin), toxin secretion (type II, type III secretion systems), motility (type IV pili, flagella), and quorum-sensing (*las, rhl, pqs*) were seen in bacteria colonizing worms compared to SK medium alone (Table 2). These transcriptomic changes suggested an acute-to-chronic virulence switch and transition from motile to sessile state during *C. elegans* colonization.

**Table 1:** Virulence factors upregulated *in vivo* (SK). Gene expression relative to that of *P. aeruginosa* on agar medium.

Gene	Fold-Change (Log2)	Adjusted *p*-value	Virulence function
*hcnA*	2.60	< 0.01	Hydrogen cyanide production
*hcnB*	2.25	< 0.01	Hydrogen cyanide production
*hcnC*	2.30	< 0.01	Hydrogen cyanide production
*algB*	1.99	< 0.01	Exopolysaccharide production
*algR*	1.33	< 0.01	Exopolysaccharide production
*algZ*	1.12	< 0.01	Exopolysaccharide production
*pelF*	3.15	< 0.01	Exopolysaccharide production
*pelG*	1.95	< 0.01	Exopolysaccharide production
*phoP*	5.47	< 0.01	Two-component sensor
*phoQ*	4.33	< 0.01	Two-component sensor
*pmrA*	4.71	< 0.01	Two-component sensor
*pmrB*	3.67	< 0.01	Two-component sensor
*pagP*	4.12	< 0.01	Lipopolysaccharide modification
*arnA*	5.80	< 0.01	Lipopolysaccharide modification
*arnB*	3.78	< 0.01	Lipopolysaccharide modification
*arnC*	4.89	< 0.01	Lipopolysaccharide modification
*arnF*	6.75	< 0.01	Lipopolysaccharide modification
*arnT*	5.70	< 0.01	Lipopolysaccharide modification
*feoA*	2.83	< 0.01	Ferrous iron uptake
*feoB*	3.61	< 0.01	Ferrous iron uptake

**Table 2:** Virulence factors downregulated *in vivo* (SK).

Gene	Fold-change (Log2)	Adjusted *p*-value	Virulence function
*pvdE*	−1.59	< 0.01	Siderophore biosynthesis
*pvdS*	−3.71	< 0.01	Siderophore biosynthesis
*fpvI*	−2.90	< 0.01	Siderophore uptake
*fpvR*	−1.93	< 0.01	Siderophore uptake
*pchA*	−3.00	< 0.01	Siderophore biosynthesis
*pchB*	−3.35	< 0.01	Siderophore biosynthesis
*pchC*	−3.35	< 0.01	Siderophore biosynthesis
*pchD*	−3.49	< 0.01	Siderophore biosynthesis
*pchE*	−3.14	< 0.01	Siderophore biosynthesis
*pchF*	−3.03	< 0.01	Siderophore biosynthesis
*pchG*	−2.56	< 0.01	Siderophore biosynthesis
*pchI*	−2.69	< 0.01	Siderophore biosynthesis
*pchR*	−3.32	< 0.01	Siderophore biosynthesis
*fptA*	−1.93	< 0.01	Siderophore uptake
*xcpP*	−1.84	< 0.01	Type II secretion
*xcpR*	−1.94	< 0.01	Type II secretion
*exoT*	−3.14	< 0.01	Secreted toxin
*exoU*	−3.14	< 0.01	Secreted toxin
*exsC*	−1.78	< 0.01	Type III secretion
*exsD*	−2.72	< 0.01	Type III secretion
*pcrG*	−3.40	< 0.01	Type III secretion
*pcrH*	−1.85	< 0.01	Type III secretion
*pcrV*	−2.65	< 0.01	Type III secretion
*pscB*	−3.94	< 0.01	Type III secretion
*pscJ*	−2.00	< 0.01	Type III secretion
*lasR*	−1.33	< 0.01	Quorum-sensing
*rhlR*	−2.16	< 0.01	Quorum-sensing
*rhlI*	−2.74	< 0.01	Quorum-sensing
*vqsR*	−1.79	< 0.01	Quorum-sensing
*pqsA*	−3.80	< 0.01	Quorum-sensing
*pqsB*	−2.44	< 0.01	Quorum-sensing
*lasA*	−1.62	< 0.01	Secreted toxin
*rhlA*	−1.68	< 0.01	Rhamnolipid production
*phzA1*	−2.91	< 0.01	Phenazine production
*phzA2*	−2.60	< 0.01	Phenazine production
*phzC1*	−3.60	< 0.01	Phenazine production
*phzC2*	−3.47	< 0.01	Phenazine production
*pilK*	−1.85	< 0.01	Motility
*pilR*	−2.23	< 0.01	Motility
*pilU*	−1.62	< 0.01	Motility
*pilZ*	−2.22	< 0.01	Motility
*flgA*	−1.38	< 0.01	Motility
*flgL*	−1.35	< 0.01	Motility
*flgM*	−2.44	< 0.01	Motility
*flgN*	−1.45	< 0.01	Motility
*fliA*	−1.26	< 0.01	Motility
*fliM*	−2.02	< 0.01	Motility

Gene expression relative to that of *P. aeruginosa* on agar medium.

*P. aeruginosa* strains from clinical samples are likely very different from the strain PA14. For better comparison, we compared our transcriptional data to a PA14 porcine *ex vivo* lung infection experiment (Harrington et al., 2022). This allowed us to understand whether the transcriptional changes of PA14 observed in our data are recapitulated in other established infection models. To compare genes that were differentially-expressed in the biofilms that colonized the lungs (analogous to SK) and the surrounding SCFM medium (mimicking the luminal mucus, analogous to growth on agar), we realigned the RNA sequencing reads to the PA14 reference genome (Supplementary Figure S1). PA14 genes upregulated in the porcine lung biofilms showed significantly greater similarity with SK conditions (Figure 4A), where one-third of DEGs were shared (n = 72/225). STRING analysis of PAO1 homologs revealed that these genes were involved in Pel exopolysaccharide, lipid A biosynthesis, and ribosomal proteins (Supplementary Figure S2; Supplementary Table S6). PA14 genes upregulated in the surrounding SCFM medium were only significantly associated with those from the *in vitro* agar condition (Figure 4B), primarily on a genomic island encoding the H3-type type VI secretion system (Supplementary Figure S3; Supplementary Table S6).

**Figure 4:**
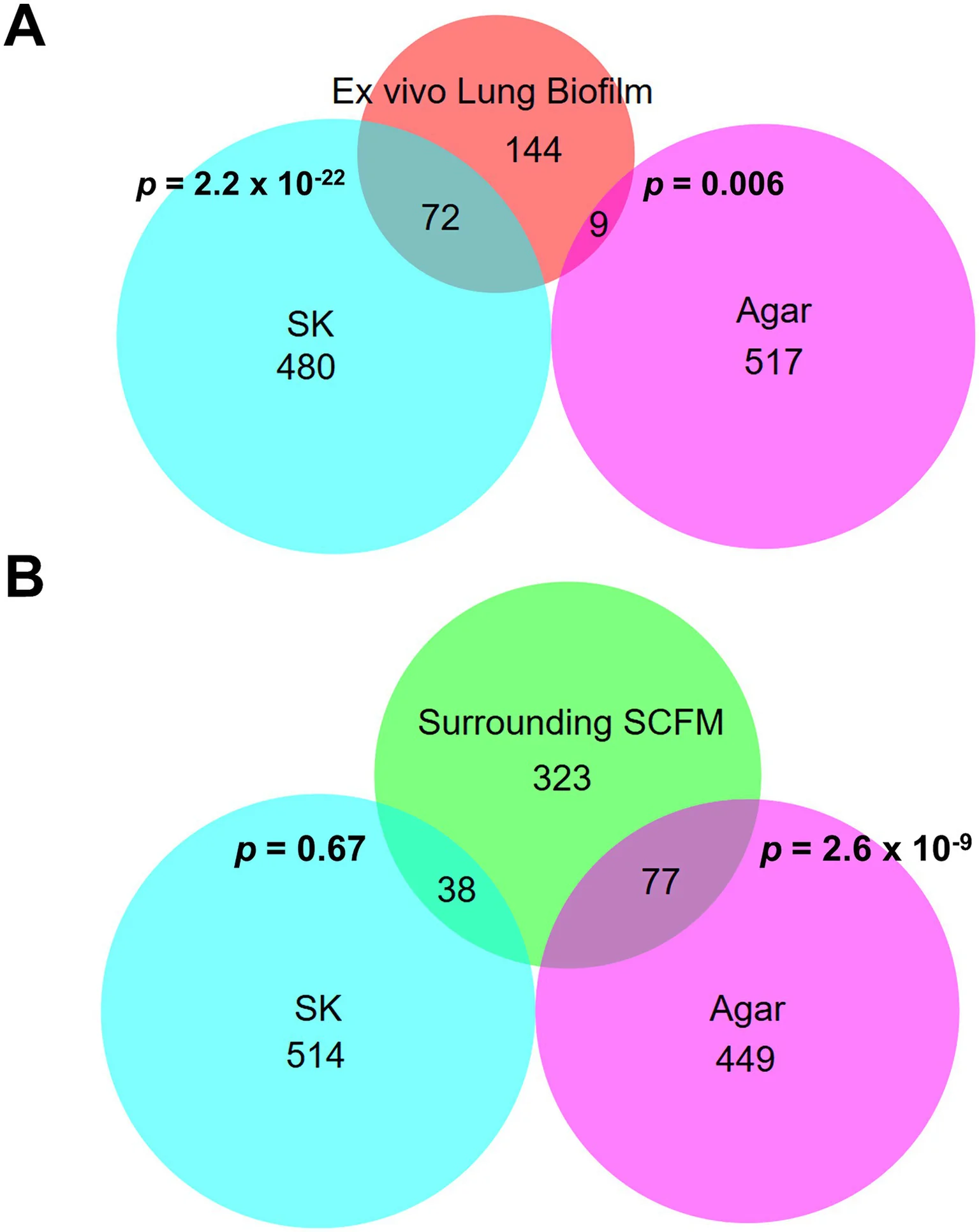
Pathways upregulated in an *ex vivo* lung biofilm model are significantly associated with SK. **(A)** Venn diagram showing the number of shared upregulated genes between the *ex vivo* pig lung biofilm model and SK or *in vitro* growth on agar. List of differentially-expressed genes from the *ex vivo* model was acquired from a previous study (24 h condition) (Harrington et al., 2022). Fisher’s exact test was performed to determine statistically significant overlap of shared genes. **(B)** Venn diagram showing the number of shared upregulated genes between *P. aeruginosa* from the surrounding SCFM medium and SK or *in vitro* growth on agar. List of differentially expressed genes from the *ex vivo* model was acquired from a previous study (24 h condition) (Harrington et al., 2022). Fisher’s exact test was performed to determine statistically significant overlap of shared genes.

Overall, transcriptomic comparisons of the *C. elegans* SK condition with those from human sputum samples and an *ex vivo* pig lung biofilm model, along with an in-depth examination of specific virulence genes differentially regulated in our RNAseq experiment, suggested that *P. aeruginosa* within the nematode host partially recapitulated chronic lung infection in humans.

### 3.3 Differentially regulated genes impact host-pathogen interactions

Finally, we explored whether *P. aeruginosa* genes that were differentially expressed during colonization of the *C. elegans* host played a role in host-pathogen interactions. We obtained in-frame deletion or transposon insertion mutants for a subset of the virulence pathways identified (*lasR, pelA, phz, PA3346, phoQ, rhlI, hsbR, pmrA, hsbA, PA2572*) and evaluated what effect mutations of these genes had on *C. elegans* Slow Kill assays. Virulence was significantly attenuated in PA14Δ*lasR,* PA14*phoQ,* PA14Δ*phz,* and PA14*rhlI* mutants (Figure 5A). To investigate these mutations’ effects on bacterial colonization, strains were transformed with a plasmid that allows constitutive expression of *dsRed* and colonization within the intestinal tract was visualized via fluorescence microscopy. Only worms infected with PA14Δ*lasR*:dsRed showed significantly decreased colonization (Figures 5B,C), as was previously shown (Tan et al., 1999a), suggesting that the other mutations likely impacted host-pathogen interactions through colonization-independent mechanisms.

**Figure 5:**
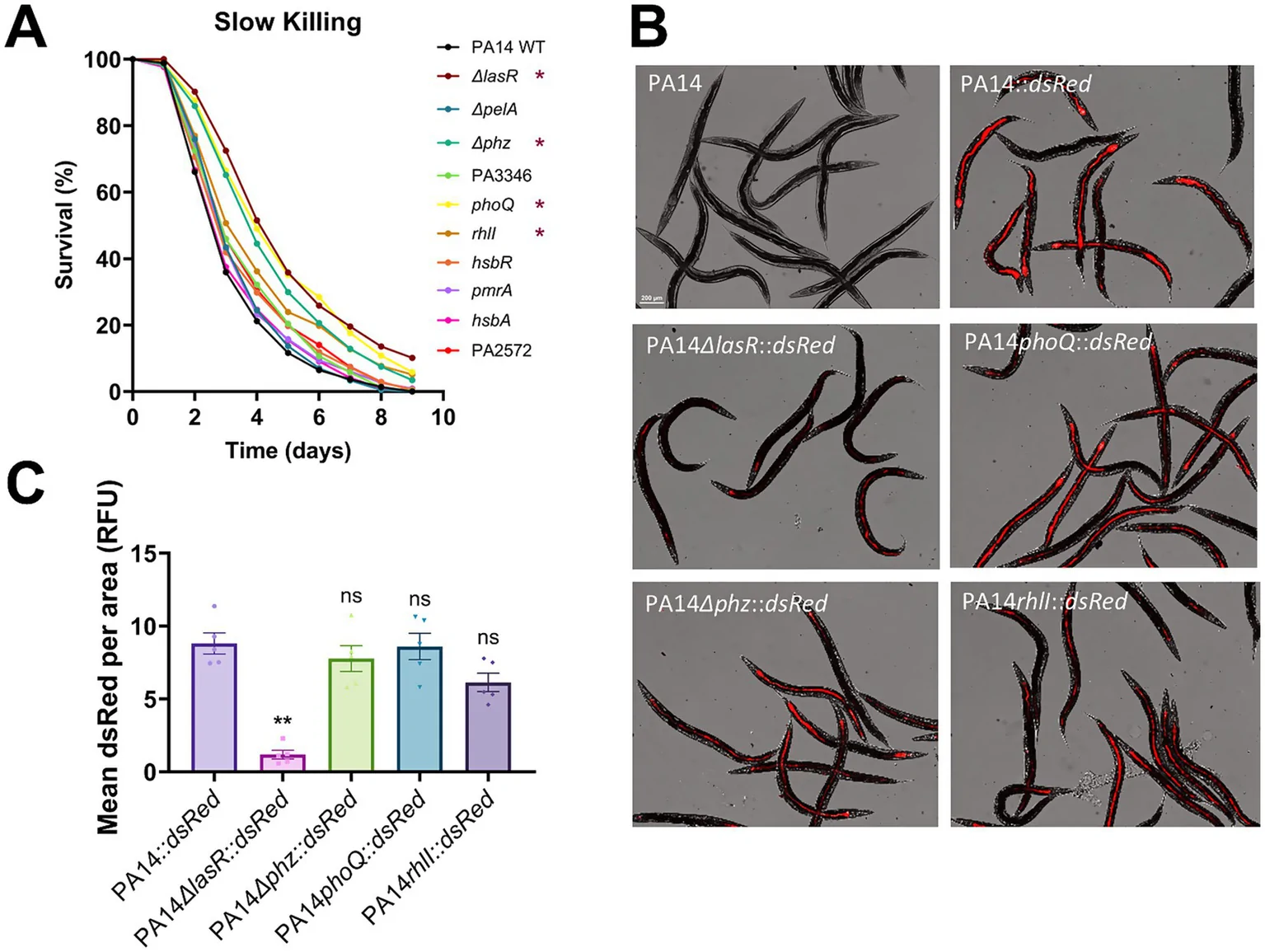
Differentially regulated genes in SK affect host-pathogen interactions. **(A)** *C. elegans* survival curves from SK assays of PA14 in-frame deletion or transposon insertion mutants. At least 50 worms per plate, three plates per condition per replicate were used. At least three biological replicates were performed. **(B)** Representative visualization of fluorescent (dsRed) PA14 colonization within the intestinal tract of worms. **(C)** Quantification of dsRed fluorescence within the worms. Five biological replicates were performed and the acquired images were analyzed via Matlab. * in **(A)** corresponds to *p* < 0.05 according to log-rank test. ** in **(C)** corresponds to *p* < 0.01 according to one-way ANOVA with Dunnett’s multiple comparisons test.

## 4 Discussion

*C. elegans* Slow Killing (SK) is a well-established model for the investigation of host-pathogen interactions in *P. aeruginosa* (Tan et al., 1999a), and has led to the characterization of innate immune pathways important for host defense (Kim et al., 2002) and virulence pathways necessary for bacterial pathogenesis (Feinbaum et al., 2012). In this study, we aimed to further characterize this model by profiling the transcriptome of *P. aeruginosa* that had colonized *C. elegans*. Within the nematode host, we observed downregulation of acute virulence determinants such as siderophore biosynthesis (pyoverdine, pyochelin), toxin secretion (type II, type III secretion), quorum-sensing (*las, rhl, pqs*), and motility (type IV pili, flagella). Interestingly, we also observed an upregulation of the *P. aeruginosa* PhoPQ and PmrAB two-component systems within the nematode host during SK. These systems are conserved across several bacterial pathogens (e.g.: *Salmonella* spp.*, Klebsiella pneumoniae*) and serve various roles in infection including the regulation of metal (magnesium and ferrous iron) uptake and modification of lipopolysaccharides (LPS) for antimicrobial peptide and colistin resistance (McPhee et al., 2006; Miller et al., 2011; Schurek et al., 2009). Hancock and colleagues utilized a different model system, human bronchial epithelial cells, to demonstrate that the PhoPQ system is upregulated during *P. aeruginosa* adherence to the epithelial monolayer (Gellatly et al., 2012). Consistently, we observed the upregulation of LPS modification genes (*pagP, arnA, arnB, arnC, arnF, arnT*) and ferrous iron uptake genes (*feoAB*) during *C. elegans* colonization.

Interestingly, investigation into specific virulence pathways revealed that intestinal colonization is not necessarily coupled to host mortality. We observed that while disruption of *lasR* attenuated *P. aeruginosa* colonization and virulence as previously reported (Tan et al., 1999a), *phoQ, rhlI*, and Δ*phz* mutants exhibited reduced virulence without impaired colonization. While the precise role of *phoQ* in virulence remains unclear, mutations in *phoQ* have been previously identified among polymyxin-resistant clinical isolates from both acute and chronic infections (Gellatly et al., 2012) and *phoQ* mutants exhibited decreased cytotoxicity against human bronchial epithelial cells (Gooderham et al., 2009). Improved host survival during infection with PA14*rhlI* and PA14Δ*phz*, both unable to produce phenazines in their strain background (Mahajan-Miklos et al., 1999; Dietrich et al., 2006), could indicate that oxidative damage by phenazine products contributes to *P. aeruginosa* pathogenesis in this model (Ray et al., 2015).

Notably, our analyses also revealed that *C. elegans* colonization partially recapitulated the transcriptomes of *P. aeruginosa* from human CF sputum samples with over 75% similarity. However, we also observed that genes involved in quorum-sensing, namely *lasR* and *rhlI*, were uniquely downregulated during SK. While this could reflect the limitations of our method of broadly comparing the transcriptomes of 24 unique clinical samples to our singular experimental condition, it may also highlight the differences between laboratory strains and clinical isolates. For instance, loss-of-function mutations in *lasR* have been readily identified in isolates from CF patients (Smith et al., 2006; Hoffman et al., 2009).

While we also attempted to profile *P. aeruginosa* during LK, where *C. elegans* is exposed to the pathogen in liquid medium instead of agar (Kirienko et al., 2013), we were unable to isolate sufficient bacterial RNA for analysis due to poor colonization (Zhang et al., 2023). However, we were able to process samples from *P. aeruginosa* grown in liquid medium in the absence of the host. This revealed that genes involved in pyoverdine biosynthesis and type II secretion were upregulated in these conditions compared to bacteria grown on agar. This was consistent with our previous reports where pyoverdine biosynthetic mutants exhibited attenuated virulence during LK but not SK (Kirienko et al., 2013), and pyoverdine-mediated iron deprivation in LK disrupted host mitochondrial function (Kirienko et al., 2015) and triggered a distinct host defense response through the Ethanol and Stress Response Element (ESRE) pathway (Tjahjono and Kirienko, 2017).

For within-strain comparisons, we also took advantage of a previous PA14 *ex vivo* pig lung infection model, where the genes upregulated in lung biofilms, such as those required for lipid A biosynthesis, were significantly associated with SK. Taken together, the *C. elegans* host appeared to at least partially recapitulate the physiological conditions of human bacterial lung infection. These data demonstrate that the SK model is an effective, efficient, inexpensive, and highly-accessible whole-organism tool to study host-pathogen interactions, including being a representative model for the human lung colonization.

## Data Availability

The datasets presented in this study can be found in online repositories. The names of the repository/repositories and accession number(s) can be found in the article/Supplementary material.
