# Supplementary material for: Caenorhabditis elegans Slow Killing recapitulates chronic Pseudomonas aeruginosa infection and reveals mediators of host-pathogen interactions
Source: Front Microbiol. 2026 Oct 21;17:1990701. doi: 10.3389/fmicb.2026.1990701 (PMC13731205; doi:10.3389/fmicb.2026.1990701)
Supplement: Supplementary file 1 [file Data_Sheet_1.PDF]

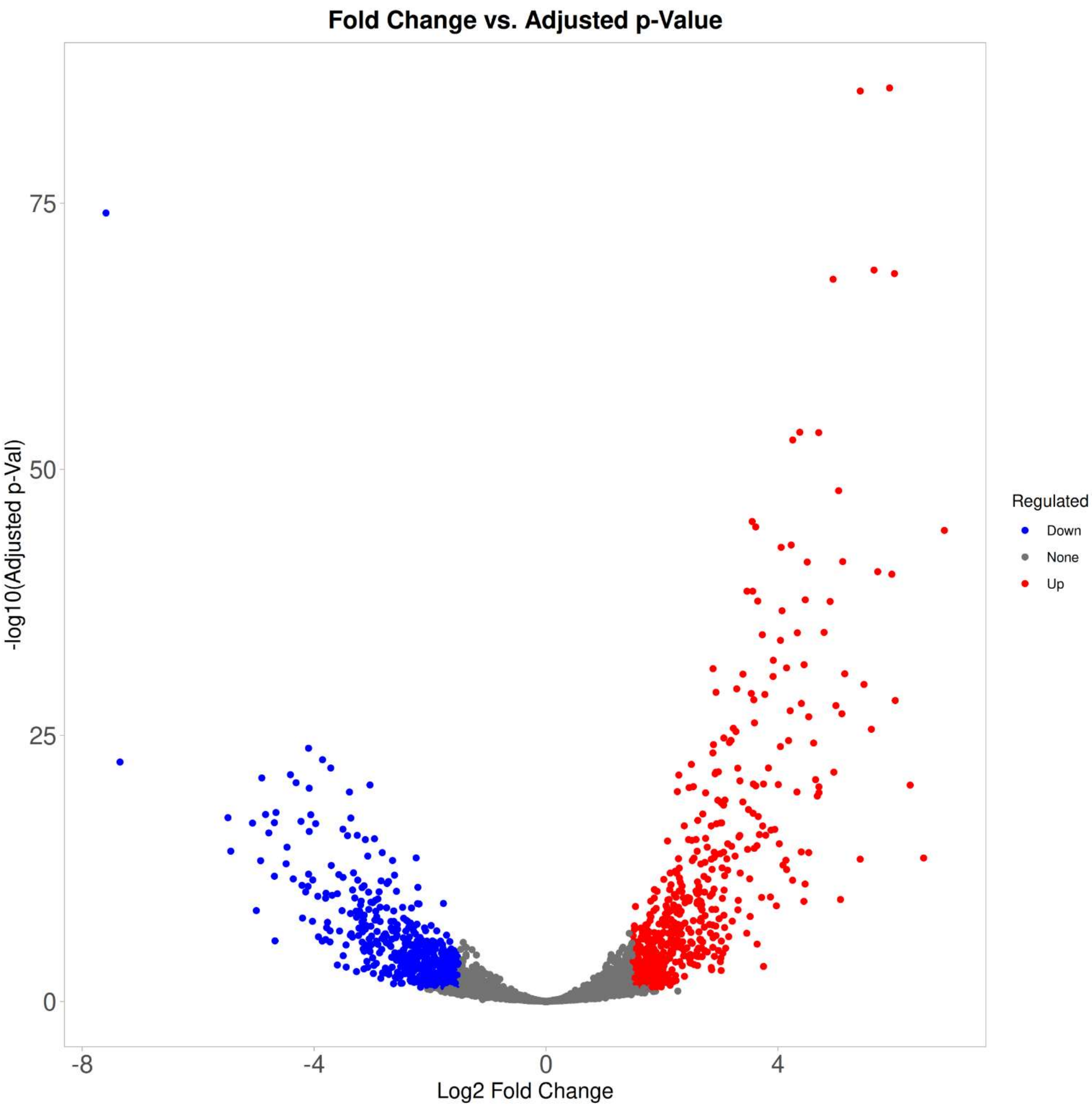

Figure S1. *P. aeruginosa* colonization in the nematode host is transcriptionally distinct from growth on agar. Volcano plot of differentially expressed genes (DEGs) derived from feature count data from sequencing reads aligned to the PA14 reference genome.

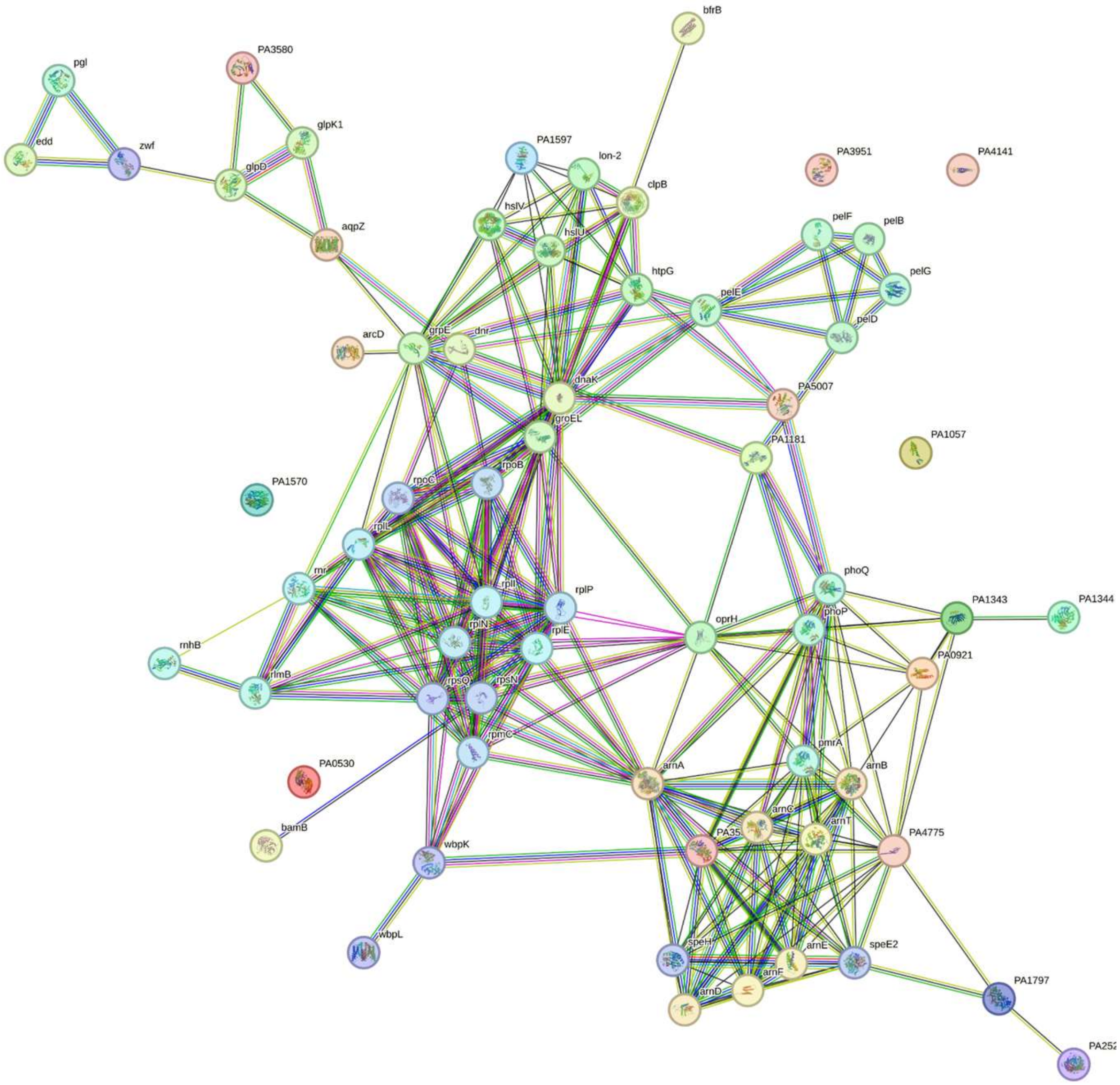

Figure S2. Upregulated genes from the *ex vivo* pig lung biofilm model share significant overlap with upregulated genes from SK. STRING analysis (based on PAO1 homologs) for the shared genes between the *ex vivo* lung biofilm and SK conditions. Enriched pathways are listed in **Supplementary Table S6**.

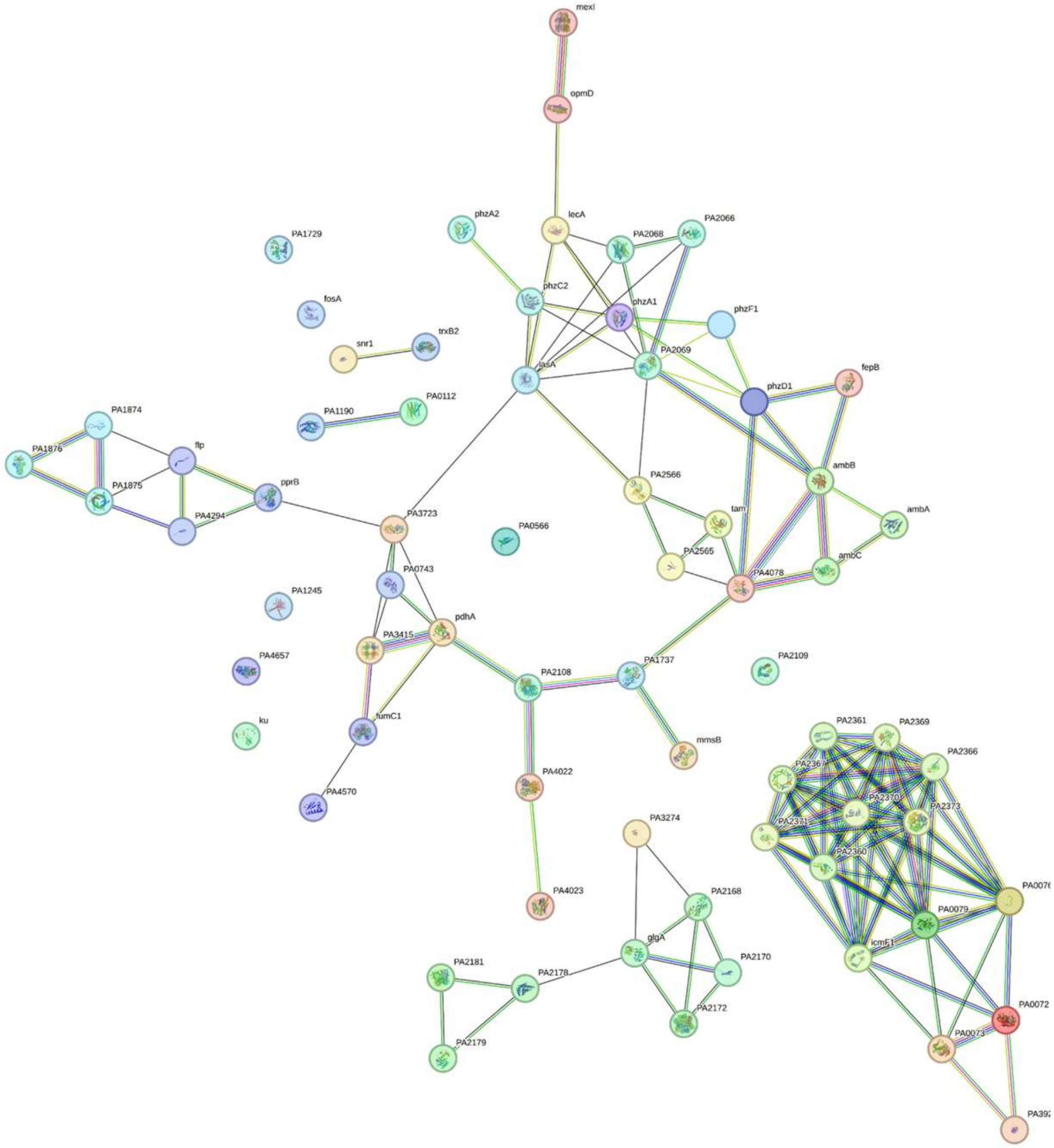

Figure S3. *In vitro* condition from the *ex vivo* lung biofilm model is significantly associated with growth on agar. STRING analysis (based on PAO1 homologs) for the shared genes between the two *in vitro* conditions. Enriched pathways are listed in **Supplementary Table S6**.
